# Acute symptomatic seizure prevention with perampanel in moderate and severe traumatic brain injury: a retrospective comparison with levetiracetam

**DOI:** 10.3389/fneur.2025.1665997

**Published:** 2025-10-16

**Authors:** Junzo Nakao, Hideki Kashiwagi, Kohei Yoshimura, Akihiro Kambara, Ryusuke Kotera, Kotaro Honda, Yu Amemiya, Junji Hatakeyama, Ken Sakakibara, Kazuma Yamakawa, Shinji Kawabata, Masahiko Wanibuchi, Akira Takasu

**Affiliations:** ^1^Department of Emergency and Critical Care Medicine, Osaka Medical and Pharmaceutical University, Takatsuki, Osaka, Japan; ^2^Department of Neurosurgery, Osaka Medical and Pharmaceutical University, Takatsuki, Osaka, Japan

**Keywords:** AMPA receptor blockade, neuroprotection, head trauma, secondary brain injury, seizure prophylaxis

## Abstract

**Background:**

Acute symptomatic seizures (ASS) occurring within 7 days after traumatic brain injury (TBI) may exacerbate secondary brain injury via excitotoxicity and elevated intracranial pressure. They are also risk factors for post-traumatic epilepsy (PTE). However, the optimal anti-seizure medication for preventing ASS remains unclear. This study aimed to compare the effectiveness of perampanel (PER) versus levetiracetam (LEV) for ASS prevention in patients with moderate to severe TBI.

**Methods:**

We conducted a retrospective cohort study including 32 patients with moderate to severe TBI who received either LEV (*n* = 19) or PER (*n* = 13) as prophylactic anti-seizure therapy. The primary outcome was the incidence of ASS within 7 days post-injury. Secondary outcomes included PTE development, psychiatric adverse events (PAEs), and functional outcomes assessed by the Glasgow Outcome Scale–Extended (GOS-E) at 3 months. Incidence rates were compared between groups using appropriate statistical tests.

**Results:**

The incidence of ASS was significantly lower in the PER group (7.7%) compared to the LEV group (42.1%) (OR 0.115, *p* = 0.050), despite a higher prevalence of cerebral contusions in the PER group. There were no significant differences in the incidence of PTE (23.1% vs. 26.3%, OR 0.84, *p* > 0.99), PAEs (23.1% vs. 26.3%, *p* > 0.99), or favorable GOS-E scores (38.5% vs. 26.3%, *p* = 0.707) between the PER and LEV groups.

**Conclusion:**

PER demonstrated a significant advantage over LEV in preventing ASS following moderate to severe TBI. Given its comparable psychiatric safety profile and functional outcomes, PER may be a promising therapeutic option for acute seizure prophylaxis in this population. However, further prospective studies with larger sample sizes are warranted to validate these findings.

## 1 Introduction

Traumatic brain injury (TBI) can lead to acute symptomatic seizures (ASS) and post-traumatic epilepsy (PTE), both of which significantly impact neurological outcomes (1, 2). ASS refers to seizures occurring within 7 days of TBI and is considered a predictive marker for the subsequent development of PTE. Therefore, preventing ASS may affect long-term prognosis (3, 4).

ASS in the context of TBI results from mitochondrial dysfunction, neuronal hyperexcitability, inflammation, and structural tissue damage (5, 6). It can exacerbate secondary brain injury by increasing cerebral metabolism and triggering glutamate-mediated excitotoxicity (7, 8). Therefore, suppressing seizures during the acute phase of TBI is not only a symptomatic intervention, but also has neuroprotective potential.

Among the current anti-seizure medications (ASMs) used for the prevention of ASS and PTE, phenytoin and levetiracetam (LEV) are most commonly administered (9); however, the evidence supporting their efficacy remains limited, and their ability to prevent either ASS or PTE are lacking (3, 4, 10–12).

In recent years, perampanel (PER) has gained attention as a selective non-competitive antagonist of *α*-amino-3-hydroxy-5-methyl-4-isoxazolepropionic acid (AMPA) receptors, which suppress excitatory neurotransmission to control seizure activity (13, 14). Beyond its antiseizure effects, PER may also exert neuroprotective properties by inhibiting neuronal cell death (15). In brain tumors, PER may not only reduce epileptiform activity, but also suppress tumor growth (16). In cerebrovascular diseases, PER demonstrates seizure control activity and is also expected to reduce infarct volume (17, 18). In TBI models, PER attenuates neuronal cell death and improves neurological outcomes, further suggesting a neuroprotective role (15, 19). These results support the possibility that PER exerts similar protective effects against ASS in TBI; however, clinical evidence regarding the use of PER for the prevention of ASS or PTE in TBI patients remains scarce. The aim of this study was to determine whether PER can reduce the incidence of ASS in the acute phase following TBI.

## 2 Materials and methods

This retrospective study included patients with moderate to severe TBI, which was defined as a Glasgow Coma Scale (GCS) score of 3–13 on admission, or those who required surgical intervention, and who were treated at Osaka Medical and Pharmaceutical University Hospital between April 2023 and March 2024. Of these patients, only those who received prophylactic administration of either LEV or PER during the acute phase of injury were included. The choice of ASM limited to LEV or PER and was made at the discretion of the attending physician. Both LEV and PER were administered intravenously for 7 days as seizure prophylaxis, and discontinued if no seizures occurred. Patients who had taken ASM before the injury or who died within 24 h following the injury were excluded from the analysis.

Our institutional protocol recommends early intravenous ASM administration in patients with GCS ≤ 13, those with traumatic intracranial hemorrhage, or those requiring craniotomy. Both LEV and PER were administered intravenously for 7 days and discontinued if no seizures occurred.

### 2.1 Definition of acute symptomatic seizures

ASS was defined as seizures within 7 days of injury. Diagnosis was made by clinical observation by physicians and nurses. Electroencephalography (EEG) was performed selectively when the level of consciousness did not correlate with neuroimaging findings or when unexplained deterioration occurred. If EEG revealed epileptiform discharges consistent with status epilepticus, the patient was diagnosed as having ASS. Seizure types were not further sub-classified in our dataset.

### 2.2 Patient selection and clinical data collection

Patients who received LEV were categorized into the LEV group, whereas those who received PER were in the PER group. Clinical data were retrospectively collected from medical records and included age, sex, mechanism of injury, GCS, and mean arterial pressure upon admission, and history of psychiatric disorders (including depression, schizophrenia, and bipolar disorder), dementia, or cerebral stroke. Additional information on the clinical course, radiological findings [acute subdural hematoma (ASDH), contusion, traumatic subarachnoid hemorrhage (tSAH)], laboratory values, and neurological outcomes was also collected.

### 2.3 Primary and secondary outcomes

The primary outcome was the incidence of ASS, which was defined as seizures occurring within 7 days post-injury. Secondary outcomes included the incidence of PTE, the incidence of psychiatric adverse effects (PAEs), and the Glasgow Outcome Scale-Extended (GOS-E) score at 3 months post-injury. A GOS-E score of 5–8 was considered a favorable outcome, whereas a score of 1–4 was considered an unfavorable outcome.

### 2.4 Statistical analysis

Statistical analyses were performed using SPSS software (version 30.0; SPSS Inc., Chicago, IL, United States). Quantitative variables were reported as the mean ± standard deviation or as median values, depending on the distribution. Categorical variables were compared using Pearson’s chi-square test, t-test, or two-tailed Fisher’s exact test, where appropriate. For continuous variables, the Mann–Whitney U test was used to compare differences between groups based on data distribution. Cumulative seizure-free survival was assessed using the Kaplan–Meier method, and comparisons between groups were made using the log-rank test. A *p*-value <0.05 was considered statistically significant.

This study was approved by the Institutional Ethics Committee of Osaka Medical and Pharmaceutical University (2841-4). The requirement for informed consent was waived because of the retrospective nature of the study. All procedures were conducted in accordance with the Declaration of Helsinki.

## 3 Results

### 3.1 Characteristics of participants

**Figure 1:**
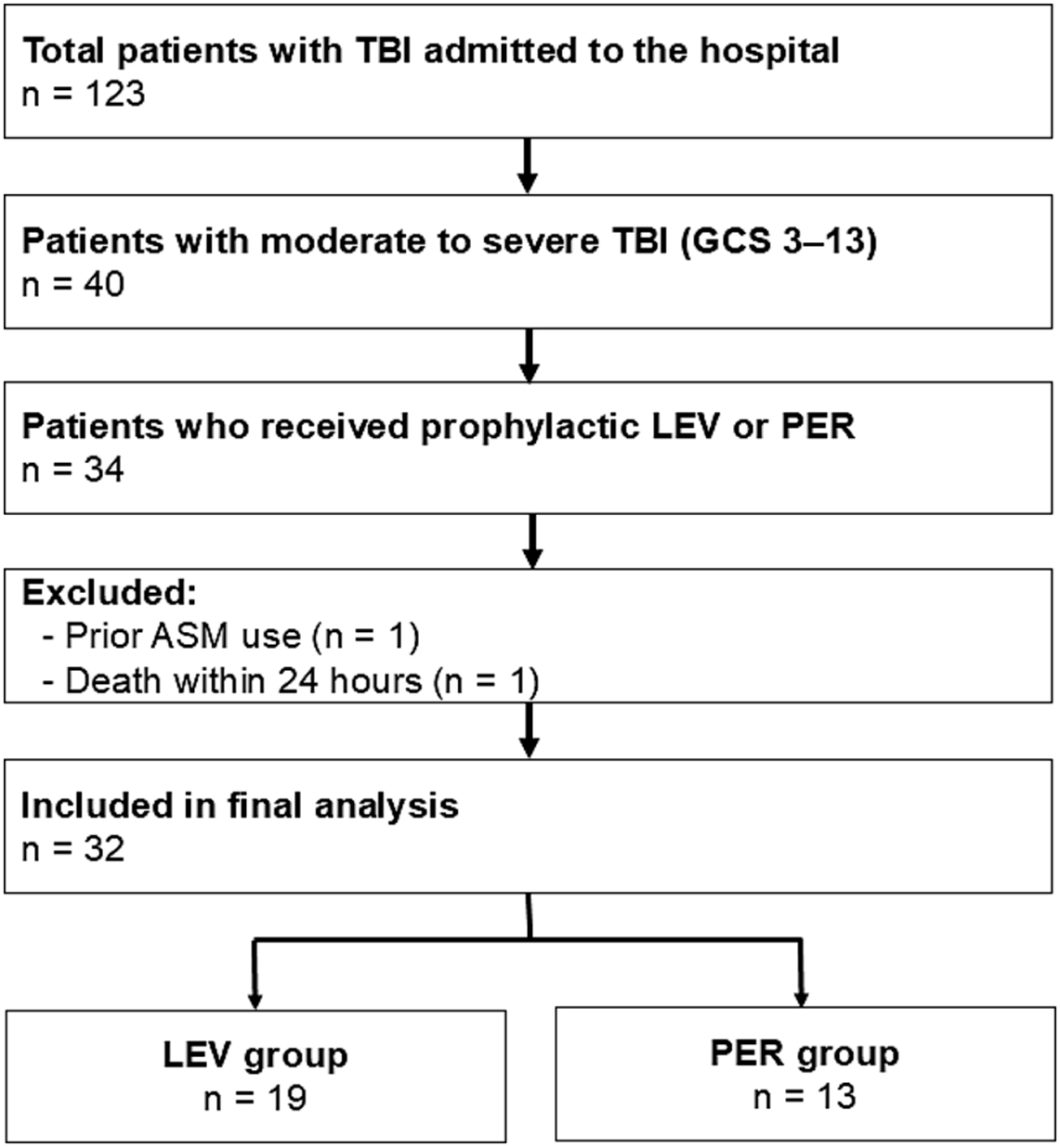
Incidence of post-traumatic epilepsy, favorable outcome (GOS-E score 5–8), and psychiatric adverse effects in the LEV and PER groups. No statistically significant differences were observed between the two groups for any outcome (PTE: *p* > 0.99, GOS-E: *p* = 0.71, PAEs: *p* > 0.99). Statistical analysis was performed using Fisher’s exact test. PTE: post-traumatic epilepsy, GOS-E; Glasgow outcome scale-extended, PAE; psychiatric adverse effects, LEV: levetiracetam, PER: perampanel.

Patients (*n* = 123) were admitted to our hospital for TBI. Forty presented with a GCS score of 3–13, and 34 received prophylactic ASM treatment during the acute phase. One patient who had been taking ASMs before injury and one patient who died within 24 h of admission were excluded. A total of 32 patients were included in the final analysis (Figure 1), with 19 receiving LEV (LEV group) and 13 receiving PER (PER group).

### 3.2 Comparison of baseline characteristics

Baseline characteristics, including age, sex, GCS on admission, past medical history, and type of injury, were comparable between the two groups, with no statistically significant differences (Table 1); however, the PER group had a significantly higher incidence of cerebral contusions compared with the LEV group (*n* = 12, 92.3% vs. *n* = 11, 57.9%, *p* < 0.05). The prevalence of ASDH and tSAH was not significantly different between the two groups (*p* = 0.06 and *p* = 0.70). The location of the contusion also did not significantly differ between groups. Except for significantly lower hemoglobin levels in the LEV group, there were no significant differences in other laboratory values between the two groups (Table 1). Overall, 29.7% of patients underwent EEG, with 36.8% in the LEV group and 30.8% in the PER group, with no significant difference between groups (*p* > 0.99).

**Table 1:** Baseline characteristics, radiological findings, and laboratory values.

Variable	LEV group	PER group	*p*-value
*n*	19	13	
Age [IQR]	74 [54–78]	64 [43–74]	0.36
Male *n*, (%)	11 (57.9)	9 (69.2)	0.71
MAP on admission (mmHg)	106.2 ± 27.6	100.4 ± 14.6	0.44
GCS [IQR]	7 [6–9]	10 [6–13]	0.18
Past history			
Psychiatric disorders *n*, (%)	3 (15.8)	2 (15.4)	>0.99
Dementia *n*, (%)	2 (10.5)	1 (7.7)	>0.99
Cerebral stroke *n*, (%)	4 (21.1)	1 (7.7)	0.63
Oral antithrombotic agent use *n*, (%)	3(15.8)	2(15.4)	>0.99
Radiological finding			
Contusion *n*, (%)	11 (57.9%)	12 (92.3%)	<0.05
ASDH *n*, (%)	13 (68.4%)	11 (84.6%)	0.06
tSAH *n*, (%)	11 (57.9%)	8 (61.5%)	0.70
Contusion location			
Frontal lobe *n*, (%)	4 (36.4)	6 (50.0)	0.14
Temporal lobe *n*, (%)	6 (54.5)	6 (50.0)	0.47
Insula n, (%)	1 (9.1)	0	>0.99
Parietal lobe *n*, (%)	0	0	
Occipital lobe *n*, (%)	0	0	
Laboratory values			
White blood cell (/μL)	11041.6 ± 496.8	11789.2 ± 3802.2	0.40
Hemoglobin (g/dL)	11.7 ± 2.4	13.3 ± 1.5	<0.05
C-reactive protein (mg/dL)	1.1 ± 2.2	0.4 ± 1.4	0.16
Fibrinogen (mg/dL)	306.9 ± 82.1	270.1 ± 50.8	0.18
D-dimer (μg/mL)	20.6 ± 15.4	34.3 ± 34.9	0.38
Albumin (g/dL)	3.7 ± 0.5	4.0 ± 0.5	0.14
Glucose (mg/dL)	161.4 ± 52.2	156.0 ± 40.3	0.70
Sodium (mEq/L)	140.5 ± 5.2	138.5 ± 3.8	0.15
Calcium (mg/dL)	8.3 ± 1.6	8.9 ± 0.4	0.38

Baseline characteristics, radiological findings and laboratory values of patients in the LEV and PER groups. Psychiatric disorders included depression, schizophrenia, and bipolar disorder. Continuous variables are presented as mean ± standard error or as median (interquartile range) for non-normally distributed data. Categorical variables are shown as counts and percentages. Comparisons between groups were performed using Fisher’s exact test or Mann–Whitney U test as appropriate. *p* < 0.05 was considered statistically significant. GCS: Glasgow Coma Scale, LEV: levetiracetam, PER: perampanel, MAP: mean arterial pressure. GCS: Glasgow Coma Scale. ASDH: acute subdural hematoma. tSAH: traumatic subarachnoid hemorrhage.

### 3.3 Incidence of acute symptomatic seizures and post-traumatic epilepsy

**Figure 2:**
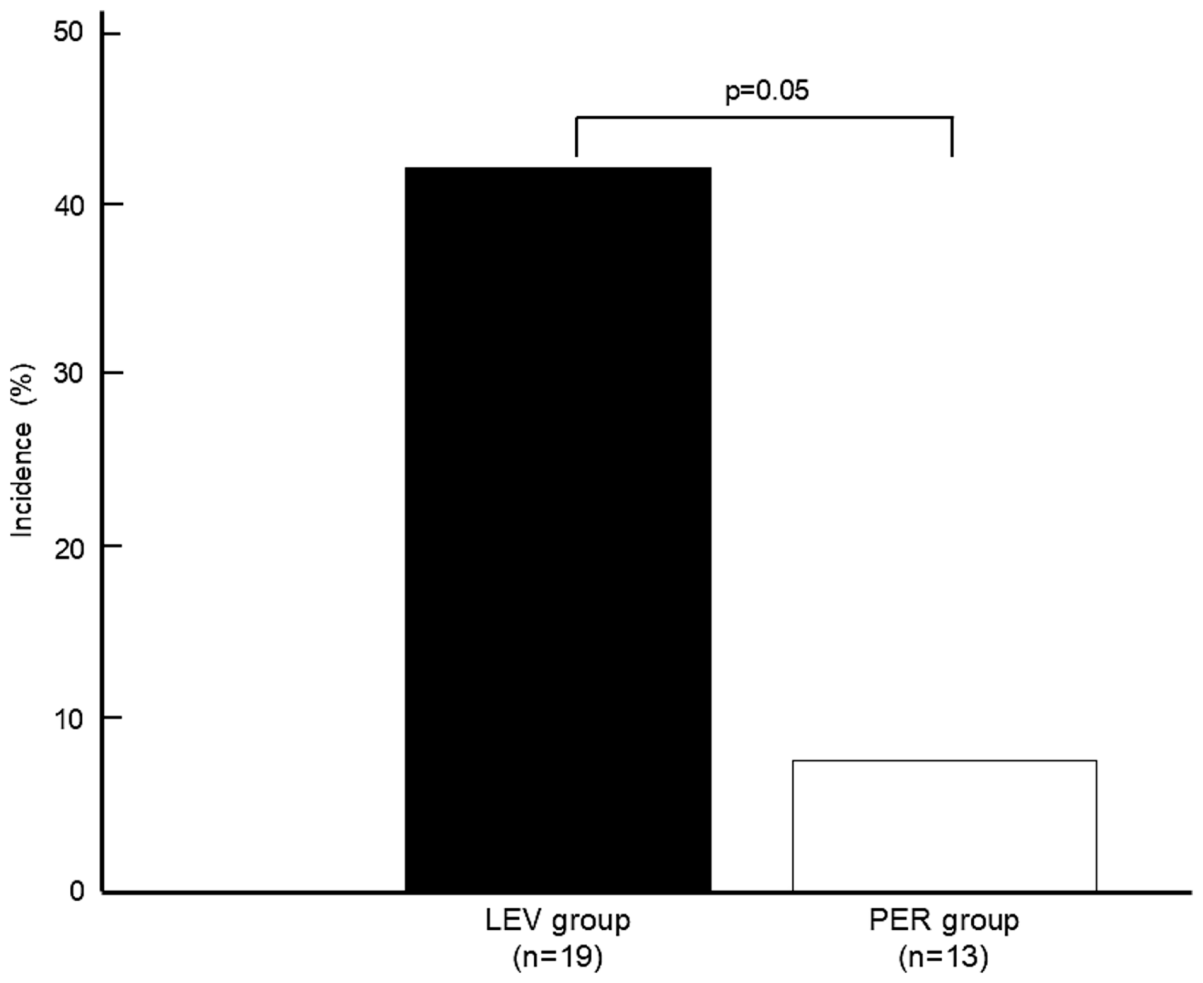
Flow diagram of the included patients. Flow diagram showing the inclusion and exclusion criteria for patients with moderate to severe TBI (GCS 3-13) who received prophylactic AED treatment. Thirty-two patients were included in the final analysis. Patients who received LEV were categorized into the LEV group, whereas those who received PER were in the PER group. TBI: Traumatic brain injury, GCS: Glasgow Coma Scale, ASM: anti-seizure medication, LEV: levetiracetam, PER: perampanel.

ASS occurred in one patient (*n* = 1, 7.7%) in the PER group compared with eight patients (*n* = 8, 42.1%) in the LEV group (OR 0.115, *p* = 0.050) (Figure 2).

**Figure 3:**
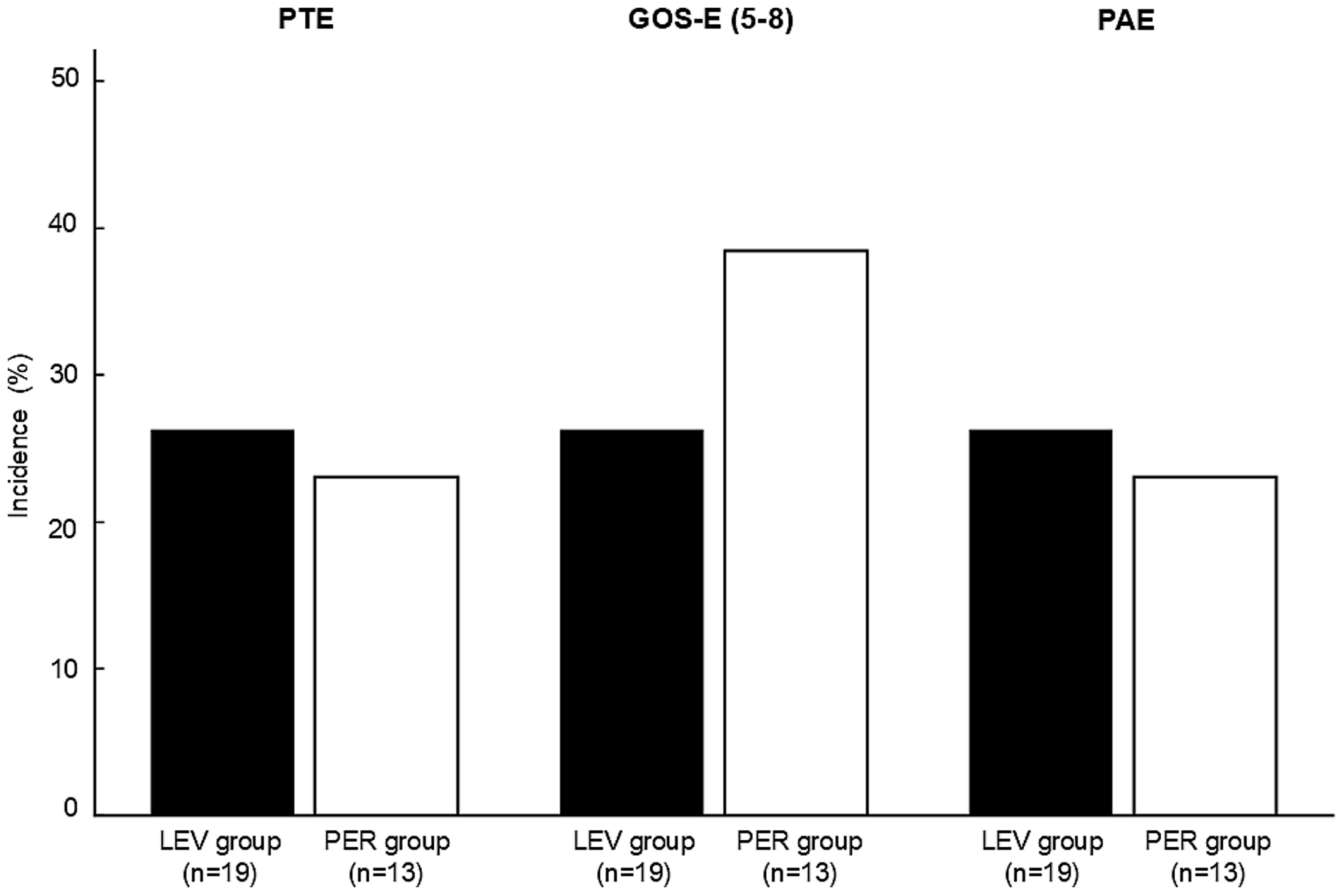
Kaplan–Meier survival curves for PTE-free survival. The cumulative incidence of PTE over 3 months from injury. No significant difference was observed between the groups (log-rank *p* = 0.62). PTE: post-traumatic epilepsy, LEV: levetiracetam, PER: perampanel.

**Figure 4:**
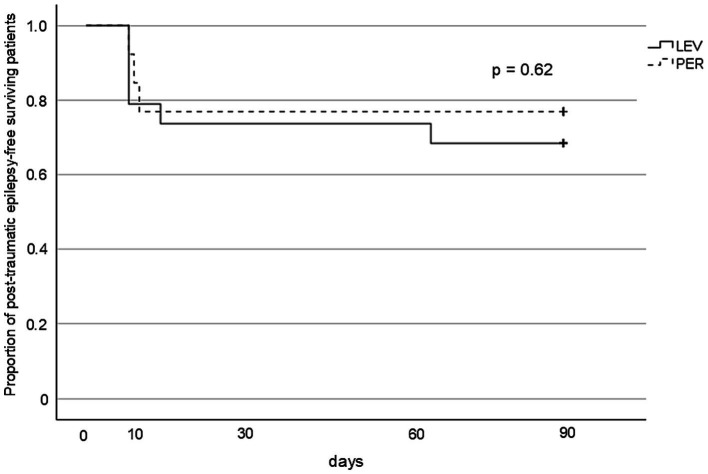
Incidence of acute symptomatic seizures. Comparison of ASS incidence between the LEV and PER groups. The PER group exhibited lower ASS rates (7.7% vs. 42.1%, *p* = 0.050). A statistical comparison was performed using Fisher’s exact test. ASS: acute symptomatic seizures, LEV: levetiracetam, PER: perampanel.

PTE occurred in five patients (*n* = 5, 26.3%) in the LEV group and three patients (*n* = 3, 23.1%) in the PER group, with no significant difference between the groups (OR 0.84, *p* > 0.99) (Figure 3). Kaplan–Meier analysis showed no significant difference in PTE-free survival within three months post-injury (*p* = 0.62) (Figure 4).

### 3.4 Functional outcomes and psychiatric adverse events

There were no significant differences between the groups in either functional outcomes [favorable GOS-E: 26.3% (the LEV group) vs. 38.5% (the PER group), *p* = 0.71] or the incidence of PAEs [26.3% (the LEV group) vs. 23.1% (the PER group), *p* > 0.99] (Figure 3).

## 4 Discussion

In the present study, we found that the incidence of ASS was lower in the PER group compared with the LEV group, despite the PER group including a significantly higher proportion of patients with cerebral contusion, which is a known risk factor for ASS (2, 20). Although the reduction in ASS did not reach statistical significance (*p* = 0.05), the observed difference between the PER and LEV groups suggests a potential clinical benefit worthy of further study. ASS exacerbates secondary brain injury through increased metabolic demand, excitotoxicity, and elevated intracranial pressure (6). Excitotoxicity through glutamate release and AMPA/NMDA receptor activation is a well-known mechanism of secondary brain injury following TBI. By inhibiting AMPA receptors, PER may mitigate these processes and reduce the occurrence of ASS (21, 22). Following TBI, alterations in the expression and function of N-methyl-D-aspartate (NMDA) and AMPA receptors have been reported (23, 24). In particular, excess activation of NMDA receptors and the resulting Ca^2+^ influx, observed shortly after the primary injury, have been implicated as pathophysiological mechanisms that drive excitotoxicity (25). The activation of NMDA receptors subsequently induces receptor phosphorylation, subunit modification, and further activation of AMPA receptors (23).

By contrast, LEV exerts its antiseizure effects through modulation of the synaptic vesicle protein SV2A, thereby regulating neurotransmitter release rather than directly targeting excitatory glutamatergic pathways (26). This mechanistic difference suggests that PER may provide unique benefits in mitigating glutamate-driven excitotoxicity that underlies ASS in the acute phase of TBI, whereas LEV provides broader synaptic stabilization. Furthermore, in a rat TBI model, PER attenuated neuronal cell death and increased anti-inflammatory cytokine expression, suggesting potential neuroprotective and anti-inflammatory effects beyond seizure prevention (27). Nevertheless, it remains uncertain whether these advantages translate into clinically meaningful superiority in acute TBI, and further prospective studies are warranted.

Because ASS is considered a downstream consequence of secondary brain injury processes, suppressing ASS with PER may not only reduce the risk of PTE, but also mitigate the extent of the secondary brain injury. Therefore, the prevention of ASS is an important component in the acute management of TBI. From this perspective, our results suggest that PER may be an effective prophylactic option for seizure control in the acute phase of TBI, particularly among patients at high risk for ASS.

Although PER appeared to effectively suppress ASS, it did not result in a significant reduction in the incidence of PTE compared with LEV (Figure 3). This discrepancy likely reflects the fundamental differences in the pathophysiology between ASS and PTE. PTE may arise from chronic epileptogenic processes that evolve over weeks to months. These include axonal sprouting, synaptic reorganization, sustained neuroinflammation, and reactive gliosis, all of which contribute to long-term changes in neuronal excitability and network structure (28, 29). Because these changes extend beyond the temporal scope of early pharmacological prophylaxis, PTE may develop despite the effective suppression of ASS.

Interestingly, several cases of PTE in the present study developed within a relatively short timeframe (Figure 4). In cases of severe TBI, prolonged inflammatory responses and persistent excitotoxicity lower the seizure threshold for ASS development (30). Moreover, such pathological neurochemical environments can last beyond 8 days post-injury (31). Therefore, in severe TBI, it may be reasonable to consider extending the risk window for ASS beyond the conventional 7-day period.

PAEs are an important concern associated with ASM. In the present study, approximately 15% of patients in the LEV and PER groups had a prior history of psychiatric disorders (Table 1). Regardless of psychiatric history, the incidence of PAEs in the PER group was comparable to that in the LEV group (Figure 3). Because of the high prevalence of psychiatric symptoms after TBI and the known psychiatric side effects of ASM (32–35), comparable PAE incidence between PER and LEV suggests that PER is a clinically acceptable alternative in this regard.

## 5 Limitations

This study had several limitations. First, the follow-up period was limited to three months, which may not adequately capture the development of late-onset PTE or long-term outcomes. Second, because of the small sample size, we were unable to perform multivariate analyses to adjust for baseline differences or potential confounders. EEG was not systematically performed in all patients, and available data only indicated whether EEG was conducted. Therefore, non-convulsive seizures may have been under detected. Third, ASM selection was based on physician discretion, which may have potentially introduced selection bias. As the choice was not randomized or blinded, residual bias cannot be excluded. Future prospective randomized and blinded studies will be required to validate our findings. Finally, this study did not assess the cost-effectiveness of each ASM, which may affect the generalizability of our findings in different healthcare settings.

## 6 Conclusion

In this retrospective study of patients with moderate to severe TBI, PER demonstrated an advantage over LEV in suppressing ASS; however, there were no significant differences between the two groups in terms of PTE, 3-month outcomes, or PAEs. Because of the established role of ASS in exacerbating secondary brain injury through excitotoxicity and increased intracranial pressure, suppression of ASS with PER may contribute to the mitigation of secondary injury processes. From this perspective, PER is a clinically viable option for seizure prophylaxis in the acute phase of TBI, particularly in patients at high risk for ASS, with psychiatric tolerability comparable to that of LEV.

## Data Availability

The raw data supporting the conclusions of this article will be made available by the authors, without undue reservation.
